# Facile synthesis of ZnMoO_4_/AlPO_4_-5 nanorod composites as visible-light-driven photocatalysts and high-performance energy storage materials†

**DOI:** 10.1039/d2ra00268j

**Published:** 2022-03-02

**Authors:** Delvin Aman, Samira Abdel-Azim, S. Said, Saad G. Mohamed

**Affiliations:** Catalysis Laboratory, Refining Department, Egyptian Petroleum Research Institute (EPRI) Nasr City 11727 Cairo Egypt; EPRI-Nanotechnology Center, Egyptian Petroleum Research Institute (EPRI) Nasr City 11727 Cairo Egypt delvin.aman@epri.sci.eg; Process Development Division, Egyptian Petroleum Research Institute (EPRI) Nasr City 11727 Cairo Egypt; Mining and Metallurgy Engineering Department, Tabbin Institute for Metallurgical Studies (TIMS) Tabbin, Helwan 109 Cairo 11421 Egypt sgmmohamed@gmail.com

## Abstract

The present article describes the facile one-step hydrothermal synthesis of single-crystalline ZnMoO_4_/AlPO_4_-5 nanorod composites. The physicochemical properties of the synthesized materials, such as structure, morphology, and bandgap, were determined using techniques such as X-ray diffraction (XRD), field emission scanning electron microscopy (FE-SEM), N_2_ adsorption–desorption isotherms, X-ray photoelectron (XPS), ultraviolet-visible (UV-vis), and photoluminescence (PL). The XRD pattern of synthesized ZnMoO_4_/AlPO_4_-5 verifies the synthesis of nanocomposites. Diffuse UV-vis spectra reveal that ZnMoO_4_/AlPO_4_-5 nanorod composites exhibit an indirect semiconductor with an optical bandgap between 3.15 and 3.7 eV depending on Mo : Zn ratio. In comparison to pure AlPO_4_-5, ZnMoO_4_/AlPO_4_-5 nanocrystal composites showed significantly higher photocatalytic activity for the degradation of *para*-nitrophenol (PNP, 0.04 g l^−1^), with 14, 99, 70, and 54% for AlPO_4_-5, Mo : Zn (2)/AlPO_4_-5, Mo : Zn (4)/AlPO_4_-5, and Mo : Zn (6)/AlPO_4_-5, respectively. This result might be attributed to the composite's efficient charge transfer and optimized electron–hole pair recombination. The supercapacitive ability of ZnMoO_4_/AlPO_4_-5 nanorod composites was also investigated in this work. For the prepared electrodes using AlPO_4_-5, Mo : Zn (2)/AlPO_4_-5, Mo : Zn (4)/AlPO_4_-5, and Mo : Zn (6)/AlPO_4_-5, the capacitance values were 400, 725, 450, and 481.25 F g^−1^, respectively, at a current density of 0.5 A g^−1^. This study shows that ZnMoO_4_/AlPO_4_-5 nanorod composites are a potential visible-light-responsive photocatalyst. The electrochemical results further demonstrate the high capacitance of ZnMoO_4_/AlPO_4_-5 nanorod composites toward energy-storage applications.

## Abstract 2

Photocatalytic degradation of PNP over single-crystalline ZnMoO_4_/AlPO_4_-5.

## Graphical abstract

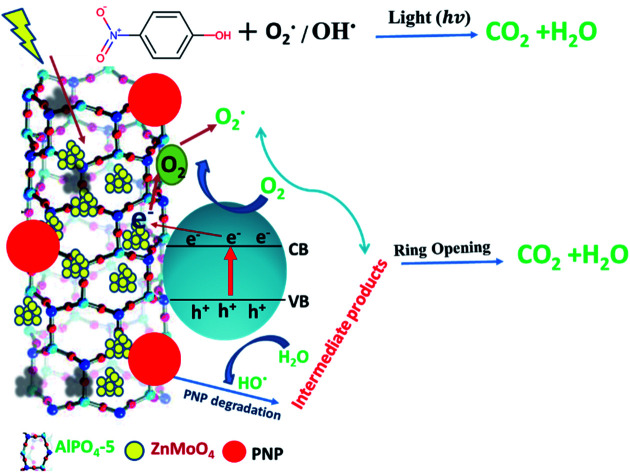

## 1. Introduction

Nanocrystalline semiconductor materials have gained increased attention because of their increased activity, high surface-to-volume ratio, and unique optical and electrical properties relative to bulk materials.^1,2^ In photocatalytic applications, titanium dioxide (TiO_2_) has received extensive research. Due to those materials' disadvantages in electron/hole recombination, their doped and composite forms have been widely synthesized and deemed successful.^3^ So, to achieve high photocatalytic efficiency cost-effectively as an alternative to TiO_2_ materials/composites, various types of semiconductor nanomaterials based on Zn were given more consideration.^4^ ZnO has been used as an effective, inexpensive, and nontoxic semiconductor to degrade a wide range of different pollutants. Due to the advantages of photocatalysis-based ZnO, semiconductors have been widely used in recent years for the photodegradation of various phenolic compounds.^5^ Although the positive attributes of photocatalysts, their applicability is limited due to the poor adsorption properties of semiconductors. Indeed, molybdenum trioxide (MoO_3_), an n-type semiconductor, can be used in enhancement visible light photocatalytic properties of ZnO semiconductor,^6^ where MoO_3_ has good structural stability, less toxicity, and low cost. Semiconductor zinc molybdate, ZnMoO_4,_ among the various types of zinc-based materials, is a particularly significant inorganic material with a wide range of applications in catalysis,^7^ electrochemical applications,^8,9^ and photoluminescence^10^ due to its superior optical and electrical properties and non-toxicity. It is an inorganic substance found in nature with two distinct crystalline phases: a-triclinic and b-monoclinic.^11^ Zinc atoms (Zn) are bound to six oxygen atoms (O), forming distorted octahedral clusters [ZnO_6_]; zinc and molybdenum atoms are bound to six O atoms which encourage the origin of distorted octahedral clusters [ZnO_6_]/[MoO_6_].^12,13^ The photoactivity of ZnMoO_4_ is a result of its structural diversity, superior optical properties in a broad emission spectrum, and substantial charge transfer between the bands of activated 2p (O_2_) and 4d (Mo^6+^) orbitals.^14^ In addition, possible improvements to the photocatalytic capabilities of traditional inorganic semiconductors could include their large specific surface area, broad visible-light absorption, and adjustable bandgap edges.^15^ It has been demonstrated that loading and dispersing ZnMoO_4_ particles on suitable supports is a viable alternative method for achieving the aforementioned goals. Various supports such as graphene oxide,^16,17^ carbon nanotubes,^18^ BiVO_4_,^19^ BiFeWO_6_,^20^ and polydopamine^21^ were studied previously to improve the loading and dispersion of semiconductor photocatalyst particles. Supports for ZnMoO_4_ photocatalyst should be developed that are inexpensive and highly efficient, such as AlPO_4_-*n* molecular sieves. AlPO_4_-*n* molecular sieves have a large surface area, uniform pores, and high thermal stability, making them ideal for use as photocatalyst supports.^22^ To our knowledge, no study has been conducted on preparing a composite photocatalyst ZnMoO_4_/AlPO_4_-5 for the degradation of harmful pollutants.

On the other hand, supercapacitors (SCs) materials have long been regarded as an attractive class of energy storage devices due to their rapid charge–discharge capability, long cycle life, safety, and reliability.^23^ Furthermore, many studies have reported that surface modification of electrode materials can lead to superior cycle and rate performance due to improved thermal and structural stability and the prevention of cation leaching.^24,25^ In ZnMoO_4_, the electrochemical contributions from both zinc and molybdenum ions are expected to be more diverse than those from a single binary oxide, resulting in improved electrochemical energy storage performance. Recently, R. Roshani and A. Tadjarodi synthesized and used ZnMoO_4_ nanoparticles as an electrode for energy storage applications.^26^ In addition, phosphide material coatings on electrode materials have shown excellent performance since the (PO_4_)^3−^ combination produces more excellent thermal stability than oxide materials.^24,25,27^ In this study, we facile fabricated ZnMoO_4_ loaded AlPO_4_ as an electrode for the first time and investigated its electrochemical performance.

Our findings have unveiled a helpful perspective for advancing inorganic composite materials with exceptional performance and improved stability for environmental purification and energy storage application. The photocatalytic performance of the ZnMoO_4_/AlPO_4_ was investigated by degrading *p*-nitrophenol (PNP) in the visible region of the spectrum light. Additionally, the other objective of this study is to develop an electrode material with high power, energy density, and cycling stability. ZnMoO_4_/AlPO_4_-5 nanocomposites, principally, demonstrated promising potential for energy storage applications.

## 2. Experimental

### 2.1. Preparation of the catalysts

Aluminophosphate was prepared by hydrothermal method with stepwise stages. The molar ratio of aluminium isopropoxide and phosphoric acid was 0.02 : 1. The trimethylamine (TEA) is used as structure directing agent (SDA). The molar ratio of the synthesis mixture gel was Al_2_O_3_ : 1.3 P_2_O_5_ : 1.2(SDA) : 205H_2_O. The gel was transferred to a Teflon lined autoclave and heated at 185 °C for one day. Then, the resultant material was washed three times and dried at 80 °C overnight. The dried sample calcined at 600 °C for 6 h in presence of dried air to remove any residue of the organic template (TEA).^7^ Incipient wetness impregnation method using molybdic acid and zinc acetate solution (purchased from Merck Company) and used without further purification to prepare ZnMoO_4_ supported on AlPO_4_-5 samples. Range of Mo/Zn molar ratios (2, 4 & 6) was used to study the suitable ratio for ZnMoO_4_/AlPO_4_-5 composites formation. The prepared samples are labeled: Mo : Zn (2)/AlPO_4_-5, Mo : Zn (4)/AlPO_4_-5 and Mo : Zn (6)/AlPO_4_-5. The resultant samples were left to sit for drying at 120 °C for 4 hours. Then the samples were calcined at 500 °C for 4 hours according to TGA analysis.

### 2.2. Characterization of the samples

The prepared samples were characterized using various techniques, including X-ray diffraction (XRD) on a Shimadzu XD-1 diffractometer using Cu Kα radiation (*λ* = 0.1542 nm) at a beam voltage of 40 kV and a current of 40 mA. The XRD peaks were indexed using the Joint Committee on Powder Diffraction Society's (JCPDS) database.

The surface area, total pore volume, and average pore diameter using N_2_ adsorption–desorption isotherms were measured using NOVA 3200 S Unite, a comprehensive automated gas sorption analyzer (Quanta chrome Corporation). Before adsorption, all samples were degassed for four hours at 300 °C in a nitrogen atmosphere to ensure a dry, clean surface. The Barrett–Joyner–Halenda (BJH) method was used to determine the pore size distributions for the desorption branch of the isotherm.

The morphology of prepared samples was tested under Field Emission Electron Microscope (FE-SEM) (Zeiss, Sigma 300VP, Germany) using an accelerating voltage of 30 kV.

X-ray photoelectron spectroscopy (XPS) was collected on K-ALPHA (Thermo Fisher Scientific, USA) with monochromatic X-ray Al K-alpha radiation −10 to 1350 eV spot size 400 μ at pressure 10–9 mbar with full-spectrum pass energy 200 eV and narrow-spectrum 50 eV.

Thermal analysis (DTC-TGA), was performed to study the structural changes of the prepared samples with thermal treatment on SDTQ-600 (TA-USA) thermo balance instrument. 10 mg of sample was heated up to 1100 °C, with a heating rate of 10 °C min^−1^ in an air flow at a rate of 100 mL min^−1^.

UV-vis spectrophotometer (Jasco model V-570) was used for recording the UV-vis spectrum with a diffuse reflectance attachment IRS-2200 (Shimadzu). Also, the spectrofluorophotometer RF-5301PC (Shimadzu) with an excitation wavelength of 300 nm was measured by photoluminescence spectra (PL).

### 2.3. Photocatalytic decomposition of *p*-nitrophenol (PNP)

The experiments were carried out using a medium pressure 125 W mercury lamp (300–460 nm, *λ*_g_ ∼ 450 nm) in a photocatalytic oxidation reactor located in the center of the cylindrical reactor. A schematic diagram of the visible light laboratory-scale photoreactor used in the current research was shown in S1.† Degeneration of *p*-nitrophenol (PNP) in an aqueous solution was evaluated at various irradiation periods. Before starting illumination, a suspension containing 1.0 g l^−1^ of catalyst and 0.04 g l^−1^ of PNP were stirred continuously in the dark for 30 min to reach an adsorption–desorption equilibrium between the surface of photocatalyst and nitrophenol molecules in the dark. The concentration of PNP in solution was used as the initial value for the kinetic treatment of the photodegradation processes. During the experiments, cooling by water circulation at 25 °C was performed at constant stirring. At illumination intervals, the sample of the solution was collected, centrifuged, and filtered through a Millipore filter (pore size 0.22 m). The filtrate was analyzed by UV-vis spectrophotometry (Shimadzu Scientific Instruments, Inc., Columbia, MD, USA). The spectral measurements were taken at a maximum absorption wavelength of PNP at 402 nm.

### 2.4. Electrochemical measurements

The electrochemical performance of the pristine AlPO_4_-5 and its corresponding ZnMoO_4_ supported AlPO_4_-5 samples: MZ (2)/AlPO_4_-5, MZ (4)/AlPO_4_-5, and MZ (6)/AlPO_4_-5 were examined as active electrode material for SC applications by the method of three-electrode cell system in 6 M KOH aqueous electrolyte solution using an electrochemical testing station (Voltalab 40 PGZ 301, Radiometer Analytical, France) at the room temperature, with a platinum wire as a counter electrode and saturated calomel electrode (SCE) as a reference electrode. The preparation of working electrodes proceeded by mixing the as-synthesized active material with carbon black, which acts as a conductive additive, and Nafion as a binder material in a mass ratio of 80 : 10 : 10, respectively. To obtain a suspended solution from the previous mixture, 0.7 mL of ethyl alcohol was added with continuous sonication. Consequently, this suspended solution was drop-casted onto the nickel foam as a current collector (1 cm (width), 2 cm (length)) (Xiamen Tob New Energy Technology Co. Ltd, China). The obtained coat was then dried at 80 °C overnight. The mass loading of the used-active material of each electrode was about 2 ± 0.5 mg cm^−2^. The electrochemical behavior for the prepared electrodes was achieved and characterized based on cyclic voltammetry (CV), galvanostatic charge–discharge (GCD), and electrochemical impedance spectroscopy (EIS) measurements.

The CV measurements were achieved within a potential range of −0.1 to 0.6 V (*vs.* SCE) at different scan rates from 10 to 200 mV s^−1^. Additionally, at varied current densities from 0.5 to 10 A g^−1^, the GCD measurements were evaluated within a potential range from −0.1 to 0.4 V (*vs.* SCE). The Nyquist plots tested the EIS measurements in the frequency range of 100 kHz to 0.01 Hz. Based on the GCD discharge curves, the specific capacitance (*C*_sp_) (F g^−1^) and specific capacity (*C*_s_) (C g^−1^) were estimated according to eqn (1) and (2), respectively.^28^1
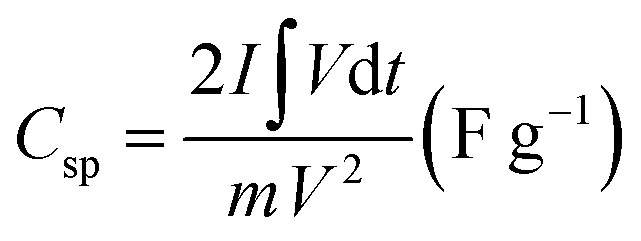
2
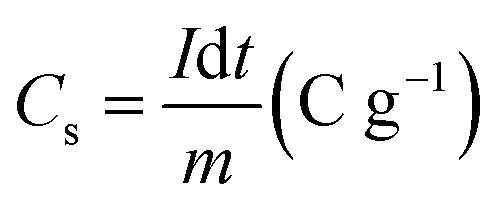
where (*I*) is the applied current (A), (*m*) represents the obtained mass of active material, (d*t*) represents the discharge time (s), (*V*) is the applied potential window, and (∫*V*d*t*) defines the integral area of voltage *vs.* discharge time for the consistent GCD-discharge curve.

## 3. Results and discussions

### 3.1. Structure and morphology

**Fig. 1:**
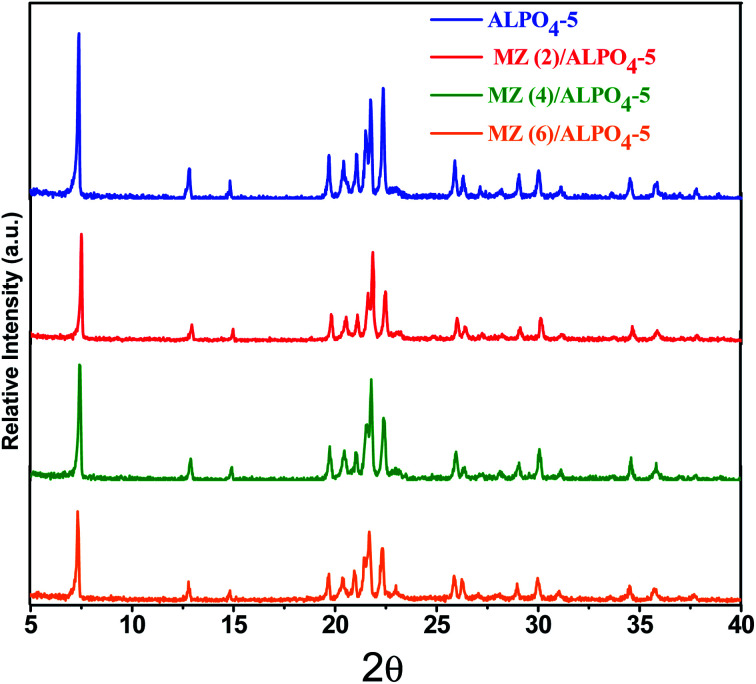
XRD spectra of Mn/Zn (*x*) AlPO_4_-5 nanocomposite *x* = 0, 2, 4 and 6.

X-ray diffraction pattern of the prepared Mo : Zn (*x*) AlPO_4_-5 nanocomposite *x* = 0, 2, 4, and 6 are represented in Fig. 1. The pattern for the AlPO_4_-5 sample identifies peaks that are sharp, narrow, and strong, at 2*θ* = 7.4, 12.84, 14.86, 19.71, 21.54, 22.38, 25.9, 29.01°. The peaks are corresponding with a hexagonal prism-shaped AlPO_4_-5 pattern (JCPDS 31-735). Else, weak and low-intensity peaks are detected at 2*θ* = 21.07, 26.3, 29.9, 34.5°, assuming AlPO_4_-5 orthorhombic structure. Furthermore, diffracted weak peaks detected at 2*θ* = 20.34, 21.78, 23.03, 35.7, are appropriate with the tridymite phase (JCPDS 36-735, dense aluminum phosphate). Therefore, the X-ray diffraction confirms that the prepared sample *via* hydrothermal method at heating temperature 185 °C for 24 h consisted of a mixture of AlPO_4_-5 and tridymite phases.

The XRD pattern for Mo : Zn (2–6)/AlPO_4_-5 samples demonstrated the phenomenon of pristine structure memorization without distortion, with well-resolved diffraction peaks related to the AFI structure, indicating that the prepared samples retain their AlPO_4_-5 crystal structure after loading and calcining. Meanwhile, as the amount of Mo : Zn ratio increases, the peak intensities of the AlPO_4_-5 phases decrease noticeably. In particular, the peak at 2*θ* = 30.5° has been slightly broadened. Since the main diffraction peak of the XRD spectrum of zinc molybdate ZnMoO_4_ is 30.52° (111) with the monoclinic structure according to (JCPDS card no: 25-1024).^4^ The broadening could be explained by a reduction in scattering contrast between pore walls and pores after the pore was covered with more molybdenum oxide in the AlPO_4_-5 support. This result is consistent with Abdelsayed *et al.*^29^ proposal that Mo species (MoO_3_) can thermally diffuse into the channels of HZSM-5 and anchor to the Brὂnsted acid sites. In general, the slight peak shift observed with MZ/AlPO_4_ catalysts is toward the lower and higher angles. The shift could result from lattice relaxation (a slight angle shift) or a strained lattice (a significant angle shift). Additionally, the presence of ZnMoO_4_ in MZ/AlPO_4_-5 catalysts results in AlPO_4_ lattice distortion, altering the interplanar distances within their crystal lattices.^30^ This result ultimately promotes good crystallinity and optical properties necessary for effective degradation/activity.

**Fig. 2:**
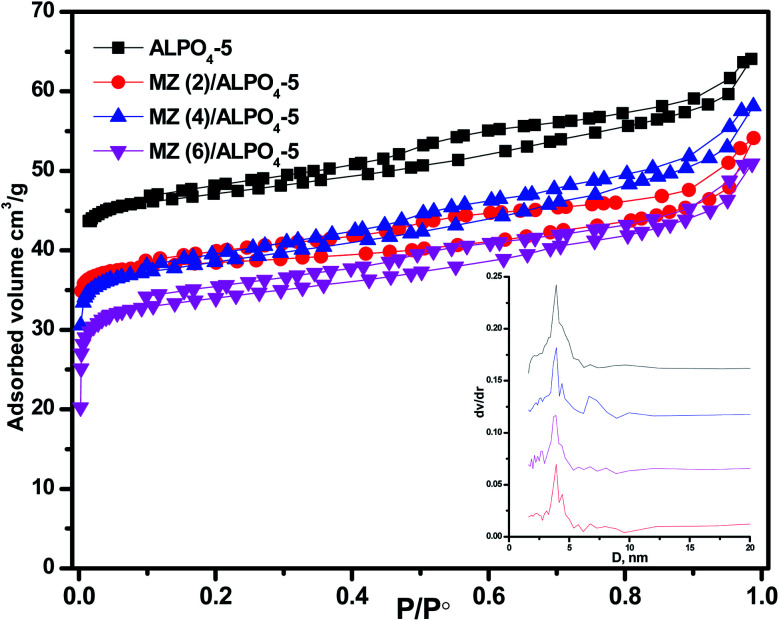
N_2_ adsorption/desorption isotherms of MZn (*x*)/AlPO_4_-5 nanocomposites, *x* = 0, 2, 4, and 6. The inset is the pore size distribution of the prepared nanocomposites.

The data on the textural parameters, the N_2_ adsorption–desorption isotherms, and the average pore size distribution curves of prepared nanocomposite samples ZM (*x*)/AlPO_4_-5 shown in Fig. 2. The nitrogen adsorption–desorption isotherms of prepared samples, which were identical in shape and did not change with loading, were classified as a mixed type (a combination of types I, II) by the IUPAC classification characteristic aluminosilicate microporous materials. According to IUPAC, the patterns of H4 hysteresis also showed slit-like mesopores particles with internal vacuum, cage-like mesopores, or partially distorted cylindrical mesoporous. H4 hysteresis loop steps show that the pore size slightly decreased (low *P*/*P*°) with Mo : Zn (2–6) loading than parent AlPO_4_-5. The BET surface area decreases AlPO_4_-5 (168 m^2^ g^−1^) > MZ (2)/AlPO_4_-5 (139 m^2^ g^−1^) > MZ (4)/AlPO_4_-5 (134 m^2^ g^−1^) > MZ (6)/AlPO_4_-5 (129 m^2^ g^−1^). Meanwhile, MZ (2–6)/AlPO_4_-5 pore diameters were nearly similar to those of the pristine AlPO_4_-5, which suggests the dispersion of metal particles occurred both on the surface and in the intermediate pristine AlPO_4_-5 layers.

**Fig. 3:**
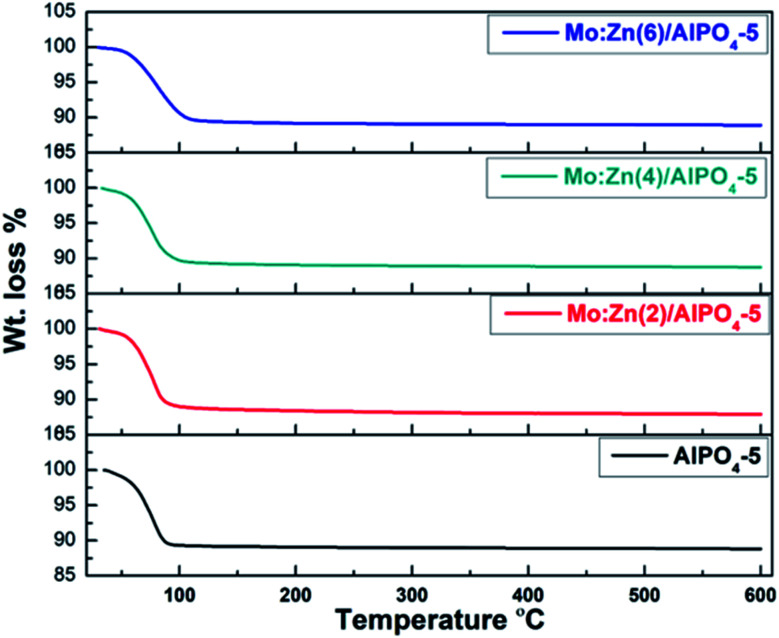
TGA curves of Mn/Zn (*x*) AlPO_4_-5 nanocomposite *x* = 0, 2, 4 and 6.

According to TGA curves of AlPO_4_-5 and Mo : Zn (2–6)-AlPO_4_-5 samples (Fig. 3). TGA analysis revealed that the prepared samples undergo two stages of weight reduction. At 60–140 °C, the initial weight loss occurs as a result of physisorbed water elimination and the onset of precursor destruction. At 120–600 °C, the second weight loss is due to the complete destruction of precursors, the dehydroxylation process, and the removal of any remaining directing agent. Indeed, when zinc and molybdenum species are co-impregnated, the overall weight loss increases slightly from 11.17 percent (AlPO_4_-5) to 12.11 percent (Mo : Zn (2)-AlPO_4_-5). By contrast, increasing the Mo : Zn mole ratio to 4 and then to 6 has no discernible effect on weight reduction. This result may be explained by the blocking of certain Zn and Mo in the AlPO_4_-5 cavities, as illustrated in BET section.

**Fig. 4:**
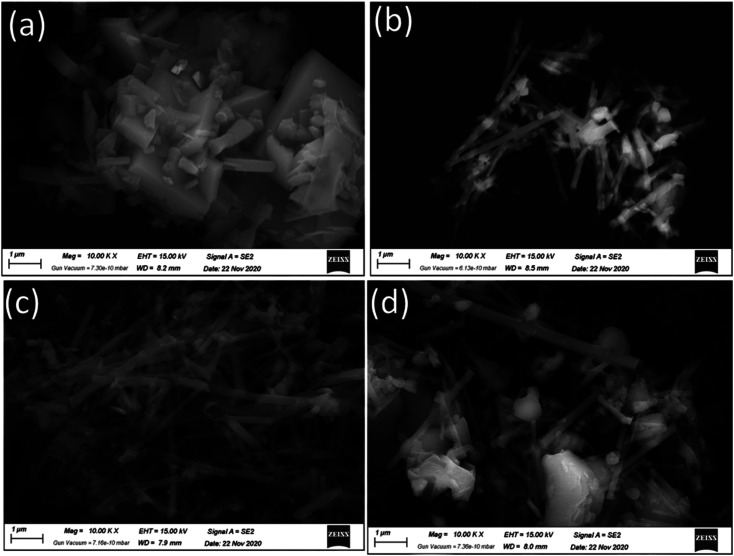
FE-SEM images of the as-prepared (a) AlPO_4_-5 molecular sieves, and (b–d)/MZ (2, 4 and 6) AlPO_4_-5 nanocomposite.

Fig. 4 shows a high-magnification FE-SEM image of the as-prepared AlPO_4_-5 molecular sieves and MZ (*x*)/AlPO_4_-5 nanocomposites nanorods, demonstrating that the nanorods are relatively uniform in size. The composites form a hierarchical nanostructure with a large amount of open space, which can be thought of as an electroactive surface. Additionally, the FE-SEM images (Fig. 3b and c) demonstrate the presence of numerous sphere-like ZnMO_4_ particles with a mean particle size of 50–100 nm highly dispersed on the surface of AlPO_4_-5 molecular sieves. That is, by using AlPO_4_-5 molecular sieves as a support, the particle size of ZnMO_4_ can be significantly reduced, thereby facilitating the photocatalytic reaction. While increasing the M : Z ratio to 6 (Fig. 3d) resulted in the formation of significant molybdenum oxide adjacent to the ZnMO_4_ nanocrystalline, as confirmed by XRD and BET.

### 3.2. XPS analysis

**Fig. 5:**
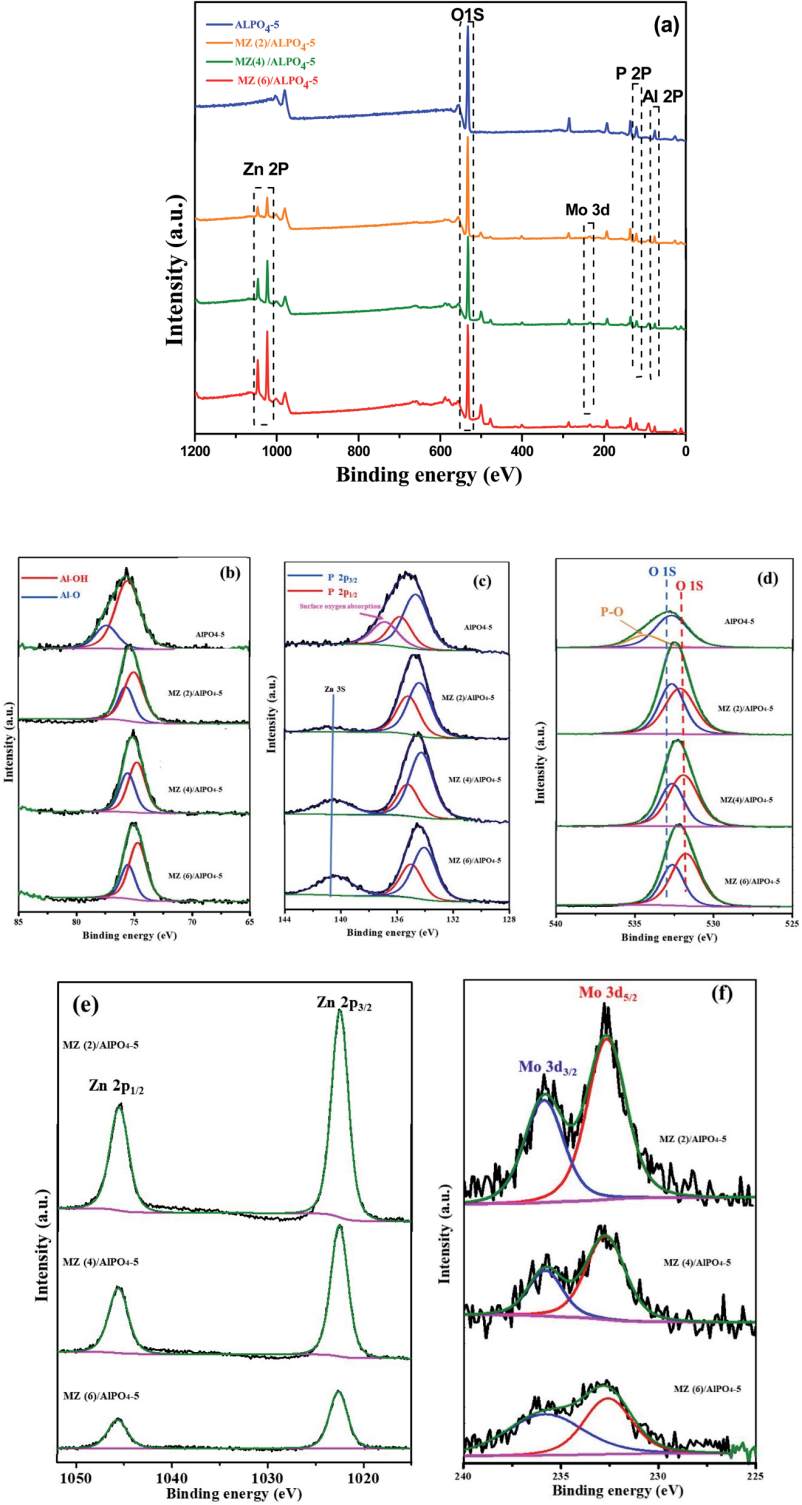
XPS spectra of Mn/Zn (*x*) AlPO_4_-5, *X* = 0, 0.1, 0.2 or 0.3 nanocomposites (a) survey of the samples, (b) Al, (c) P, (d) O, (e) Zn and (f) Mo spectra.

To examine the surface elemental composition and different valence states of doped molybdenum and zinc in MZ (*x*)/AlPO_4_-5 *x* = 0, 2, 4, or 4 catalysts, XPS was carried out (Fig. 5). The XPS survey spectrum (Fig. 5a) showed the presence of Al (2p), P (2p), O (1s), Mo (3d_5/2_, 3d_3/2_), and Zn (2p) in the prepared catalysts. Additionally, the Al (2p) peak (Fig. 5b) is not observed at a binding energy of 74 eV, implying the absence of Al_2_O_3_.^31^ The O 1s spectrum shown in (Fig. 5d) also indicates a change in the chemical state of O in MZ (2–6)/AlPO_4_-5 nanocomposite compared to bare AlPO_4_-5. Further, the high-resolution O 1s spectra shown are composed of the lattice oxygen species (O 1s) at 530.0 eV and surface adsorbed oxygen ions (O_ads_) at 532.5 eV,^9^ which could be assigned to oxygen in metal oxide for ZnO and MoO_3_ species. The Zn_2p_ XPS spectrum (Fig. 5e) showed binding energy peaks at 1022.7 eV and at 1045.5 eV assigned to Zn (2p_3/2_ & 2p_1/2_) of the Zn^2+^ state. The intensity of the peaks decreases with the loading of Mo increased. Also, metallic Zn with a binding energy of 1021.50 eV was not observed, which confirmed that Zn exists only in the oxidized form.^32^ The elements individual (XPS) regarding Mo 3d in prepared samples depicted in (Fig. 5f) shows two well-resolved spectral peaks at the binding energy region of the Mo 3d ranging from 232.96 to 236.17 eV due to Mo^6+^ 3d_3/2_, Mo^6+^ 3d_5/2_, respectively.^9^ Thus, molybdenum oxides are very close to existing as MoO_3_ phase in agreement with the XRD results.^33^ The XPS results confirm that in the ZnMoO_4_ nanocrystals obtained; there are valence states of +2, −6, and −2, respectively. It was noticed that by increasing Mo : Zn molar ratio while maintaining the Mo content constant, the XPS peak intensity decreased by increasing the Mo content concentration up to Zn : M (6)/AlPO_4_-5 catalysts. This finding suggests that bigger molybdenum oxide particles could be formed on the outer surface of AlPO_4_-5 support (matching BET results) may be due to the possible migration of molybdenum oxides into AlPO_4_-5 channels during the calcination step.

### 3.3. Optical properties

**Fig. 6:**
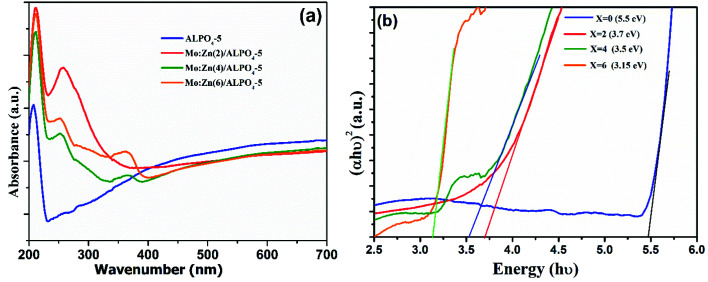
UV-Vis diffuse reflectance spectra (a) and (*αhv*)^2^*vs.* hυ curves (b) of AlPO_4_-5 molecular sieves, and MZ (2, 4 and 6) AlPO_4_-5 nanocomposites.

It is well known that UV-vis diffuse reflectance spectroscopy is a vital characterization technique for semiconductor photocatalysts. The semiconductor optical properties have their origin in both intrinsic and extrinsic effects. Intrinsic optical transitions occur between the electrons in the conduction band and holes in the valence band caused by light excitation. In contrast, extrinsic properties are related to dopants/impurities, which usually create electronic states in the bandgap, which results in the dissipation and variation of optical parameters.^34^ The UV-vis diffuse reflectance spectra of the as-prepared AlPO_4_-5 and MZ (2, 4 and 6) AlPO_4_-5 nanocomposite were measured within the 200–700 nm region, and the results are shown in Fig. 6. It can be seen clearly that the pure AlPO_4_-5 molecular sieves are white powder and show no absorption in the range of visible light. However, after loading ZnMoO_4_ with different M/Z ratios over AlPO_4_-5 molecular sieves, the composite material exhibited absorption in the visible light range due to the transition between the valence and conduction band (Fig. 6a). Where the absorption was valued to 460, 365, and 360 nm for Mo : Zn (2)/AlPO_4_-5, Mo : Zn (4)/AlPO_4_-5 and Mo : Zn (6)/AlPO_4_-5 catalysts, respectively. Thus, Mo : Zn (2)/AlPO_4_-5 catalyst has the highest absorbance with *λ*_g_ at 460 nm.^35^ Moreover, the absorption intensity slightly decreased for the MZ (2, 4, and 6) AlPO_4_-5 nanocomposite, increasing the M/Z ratio. The above results indicate that MZ (2, 4, and 6) AlPO_4_-5 nanocomposite are potential visible-light-driven photocatalysts.

The reflectance data were transformed using the Kubelka–Munk function (3)^36^ to determine the gap energy. Thus, using the *F*(*R*) function in conjunction with the Tauc eqn (4), the spacing between the conduction band and the valence band of the synthesized material was estimated as follows:3
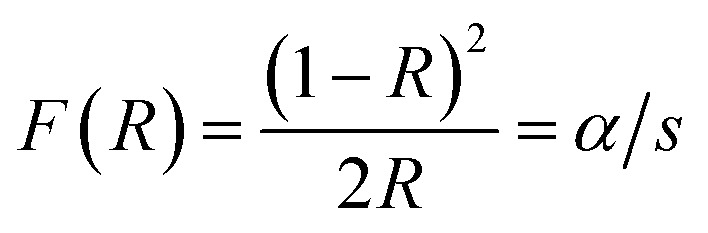

4 (*αhv*) = (*hv* − *E*_g_)^*n*^

where *α* is absorption coefficient directly proportional to *F*(*R*), *hv* and *E*_g_ are photon energy and optical band gap energy, respectively, and *n* equals 1/2 because ZnMoO_4_ is an indirect gap semiconductor.^30^

The extrapolation of the straight line to (*αhν*) = 0 gives the gap energy value. The estimated values for *E*_g_ of the samples in this work are between 3.15 and 3.7, as observed by linear region extrapolation. The obtained values are very consistent with those found in other studies. Zhang *et al.*^36^ determined the bandgap energy of α-ZnMoO_4_ synthesized electrochemically to be 3.64 eV. Some factors may interfere with the value of *E*_g_, such as processing techniques structural characteristics (degree of crystallinity, mean particle size, lattice parameter values). A band around 3.15 eV (∼390 nm) is observed in Fig. 6; this characteristic is associated with defects present in the ZnMoO_4_ lattice due to high concentration molybdenum than zinc. The results indicate that the higher photocatalytic activity of Mo : Zn (2)/AlPO_4_-5 catalyst was due to the synergistic effect of Mo and ZnO in the presence of AlPO_4_-5 zeolites. While the incorporations of Mo-dopant on ZnO lead to a blue-shift in the optical gap energy.^37^ These incorporations create shallow donor states partially filled in the bottom of the conduction band around Fermi level.^38^ Furthermore, the difference between the valence electrons of Mo^6+^ and Zn^2+^ is 4, which can produce enough free carriers.^39^ Also, Mo has multiple valence states of +6, 5, 4, 3, 2,^40^ which suggests that each Mo atom can contribute 3 or 4 free electrons depending on valency to the ZnO lattice that can modify optical absorption and emission processes of ZnO. On the other hand, the presence of AlPO_4_-5 zeolites, which possess electrons rich sites and then can donate an electron to ZnO semiconductor molecules.

**Fig. 7:**
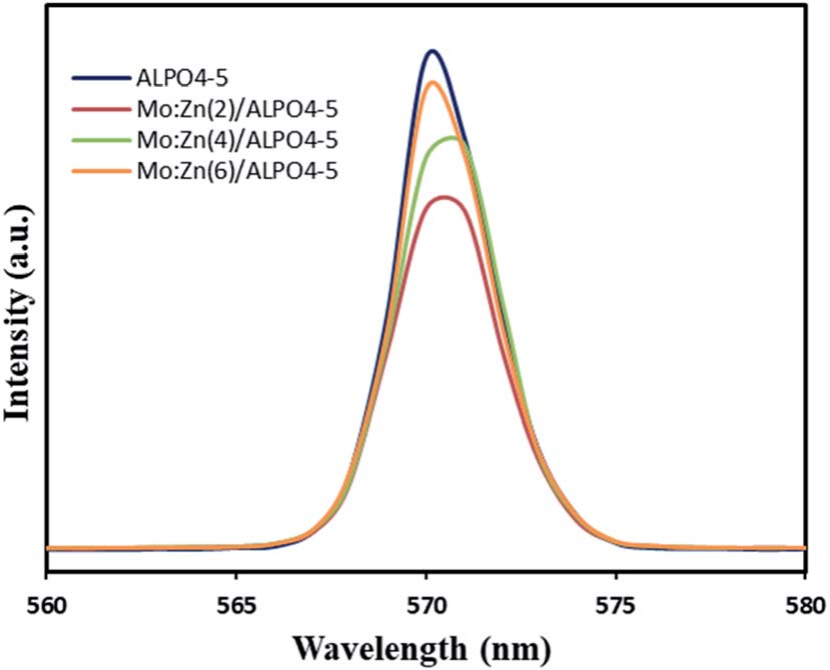
PL spectra of AlPO_4_-5 molecular sieves, and MZ (2, 4 and 6)/AlPO_4_-5 nanocomposite.

The photocatalytic efficiency is mainly controlled by the separation and migration of the photo-generated charge carriers because if the electron–hole pairs recombine during the photocatalytic process, a significant amount of photonic energy is lost. Therefore, it is vital to suppress the recombination of charge carriers to increase the catalyst's activity. Thus, the photoluminescence (PL) spectra have been used to investigate carrier trapping and charge carriers' recombination. Fig. 7 shows the photoluminescence (PL) obtained for bare AlPO_4_-5, Mo : Zn (2)/AlPO_4_-5, Mo : Zn (4)/AlPO_4_-5 and Mo : Zn (6)/AlPO_4_-5 catalysts, measured in the range of 500–600 nm at an excitation wavelength of 300 nm. All the prepared catalysts showed one broad emission peak; a well-defined peak was observed in the spectrum at 570 nm, which can be attributed to the inter-band (CB–VB) radiation recombination. This main peak is a significant and characteristic emission peak for recombination of catalyst, which emits light with energy equal to or slightly greater than the bandgap of photocatalysts. We observed that bare AlPO_4_-5 shows a strong peak at 570 nm, and the position of this peak is not affected by doping of the bare AlPO_4_-5 with any amount of Mo : Zn. But the intensity of this emission peak is significantly reduced with Mo : Zn doped AlPO_4_-5, where these metal oxides can function as agents to capture photo-produced electrons.

### 3.4. Photocatalytic activity

**Fig. 8:**
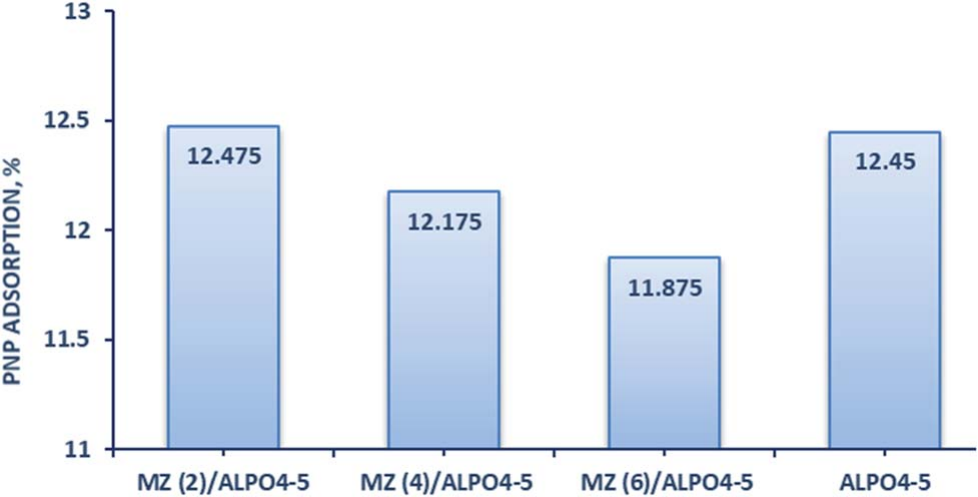
The adsorption ability of prepared catalysts for PNP degradation after 30 min in the dark.

To evaluate the adsorption ability of bare AlPO_4_-5, Mo : Zn (2)/AlPO_4_-5, Mo : Zn (4)/AlPO_4_-5, and Mo : Zn (6)/AlPO_4_-5 catalysts for degradation of PNP solution was run under 30 min dark condition, shown graphically in (Fig. 8). The high adsorption capacity of all catalysts in the dark can be attributed to their high surface area and porosity. It can be clarified that the adsorption process depends mainly on the surface area of the adsorbent. Since AlPO_4_-5 has the highest surface area (168 m^2^ g^−1^) and thus gave an adsorption capacity (12.45%). On the other hand, Mo : Zn (2)/AlPO_4_-5, Mo : Zn (4)/AlPO_4_-5 and Mo : Zn (6)/AlPO_4_-5 catalysts have less surface areas (139 m^2^ g^−1^, 134 m^2^ g^−1^ and 129 m^2^ g^−1^, respectively) but gave the similar adsorption capacity compare with unloaded AlPO_4_-5 (12.475, 12.175 and 11.875, respectively). The adsorption of PNP on the surface of the AlPO_4_-5 sample could occur *via* surface adsorption and electrostatic attraction between the protons of PNP and the negative charge on the AlPO_4_-5 surface in the present study.^41^ PNP is slightly acidic in solution and AlPO_4_-5 is composed of AlO_4_ and PO_4_ rings connected *via* oxygen bridges. Indeed, the formation of ZnMoO_4_/AlPO_4_-5 composites with varying MZ ratios can give a negative charge and basicity to the AlPO_4_-5 framework and change the porosity of the resultant composite be advantageous for boosting PNP adsorption. Additionally, the increased amount of PNP adsorbed by MZ ratios may be due to the fact that MZ cations increase the basicity of the composite surface, increasing its attraction for acidic molecules such as PNP.^42^ Also, the synergistic effect of ZnMoO_4_ and the AlPO_4_-5 matrix provided the MZ/AlPO_4_-5 composite with its adsorption capability.^43^ Instead, the MZ ions may act as unique adsorption sites, presumably by covalently interacting with the PNP molecule's benzene ring. Plus, as the number of active sites (Zn and Mo) on the composites surface increases, the electron transfer rate and adsorption of the PNP molecules increase, which are perfect properties as photocatalyst and supercapacitor.^42,43^

**Fig. 9:**
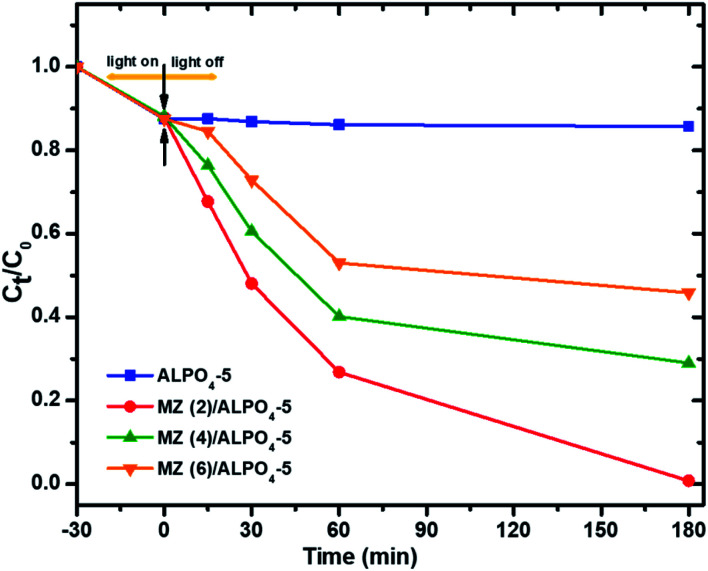
Photocatalytic degradation of PNP over prepared catalysts. Reaction conditions: the dosage of catalysts 1.0 g l^−1^, initial PNP concentration of 0.04 g l^−1^ after 180 min irradiation time.

The photocatalytic performances of AlPO_4_-5, Mo : Zn (2)/AlPO_4_-5, Mo : Zn (4)/AlPO_4_-5, and Mo : Zn (6)/AlPO_4_-5 catalysts were tested by the degradation of PNP. As shown in Fig. 9, in the presence of Mo : Zn semiconductors in the AlPO_4_-5 zeolite catalysts, the degradation activity becomes much higher than the unloaded AlPO_4_-5 zeolite catalyst. It is found that a degradation percentage of PNP was attained over current prepared zeolite-based photocatalysts, 14.3, 99.25, 70.09, and 54.15% with unloaded AlPO_4_-5, Mo : Zn (2)/AlPO_4_-5, Mo : Zn (4)/AlPO_4_-5 and Mo : Zn (6)/AlPO_4_-5, respectively after 180 min irradiation time. This result can be explained as during the photoexcitation of Mo : Zn molecules can eject an electron which delocalizes through a framework or in clusters of charge balancing cations. Also, there are reverse processes in which zeolite-rich electron sites can donate an electron to Mo : Zn photoexcited molecules. Thus, the transferred photo-induced electrons participate in the oxidative degradation of organic pollutants, which leads to enhanced photocatalytic activity of the prepared zeolite-based photocatalysts. Where zeolite has several specific features that make them suitable as a host for photocatalyst,^44^ because of its inherent superior photocatalytic properties, Mo : Zn (2)/AlPO_4_-5, which contains the highest percentage of ZnO semiconductors, was found to have the highest photocatalytic activity. Also, this can be interpreted that in the case of the use of catalysts that have high Mo : Zn loading on AlPO_4_-5 zeolite, particles may tend to aggregate, reducing the interface between the reaction solution and the photocatalyst, decreasing the number of ZnO active sites on the catalyst surface by masking some parts of the photosensitive surface and consequently hinder or even reflect light penetration.^45,46^ Hence, Mo : Zn (2)/AlPO_4_-5 has to be added to confirm the total absorption of light photons for efficient photo-mineralization. This result agrees with XRD data in which successive increase in Mo : Zn mole ratio from 2, 4, to 6 causes a noticeable decrease in the peak intensities of AlPO_4_-5 and tridymite phases. This result significantly reflects the covering of Zn particles onto the surface and strong interactions between Mo species and the AlPO_4_-5 framework, leading to lower crystallinity. Furthermore, the XPS spectra of the synthesized samples represented that the co-impregnation of Mo : Zn species on AlPO_4_-5 affected the intensity of the XPS bands. As clarified, the band intensity was increased with the increase of Mo : Zn molar ratio from 2 to 4; meanwhile, it increases at molar ratio 6. Moreover, the Mo : Zn (2)/AlPO_4_-5 possesses oxygen-containing groups (O–Al–O, O–P–O, P–O–Zn and chemisorbed H–O–H molecules). These oxygen-containing groups may either recombine or interact with its charge carriers to produce reactive oxygen species.^47^ Therefore, these reactive oxygen species are responsible for breaking the compound's chemical bonds and could cause the complete mineralization of organic compounds.^48^

### 3.5. Kinetic fitting

**Fig. 10:**
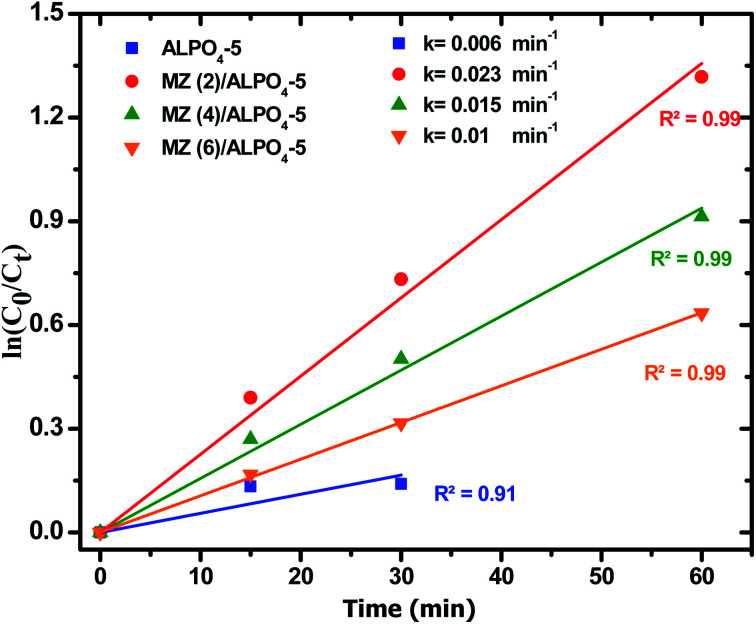
First order plots of PNP degradation over prepared catalysts. Reaction conditions: the dosage of catalysts 1.0 g l^−1^, initial PNP concentration of 0.04 g l^−1^ after 180 min irradiation time.

Moreover, the regression curve of the natural logarithm of normalized PNP concentration *versus* reaction time is straightly linear, indicating that the kinetics of PNP degradation over these photocatalysts can be considered as simple first order as expressed in eqn (5):5
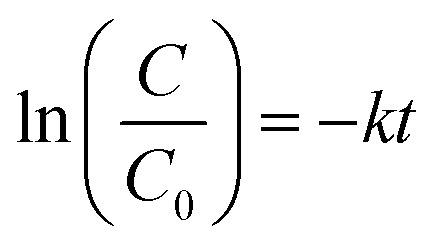
where *C* is the concentration of PNP (g l^−1^) at time *t* (min), *C*_0_ is the concentration of PNP (g l^−1^) at *t* = 0 (min), and *k* is the reaction rate constant (min^−1^). The rate constants (*k*) were calculated from the slopes of the straight-line portion of the plots of ln(*C*/*C*_0_) *versus t* as a function of the used experimental parameters. The kinetics for the photodegradation PNP after 30 min irradiation time is shown in (Fig. 10), where *k* can be determined from the slopes of the linear plots. It was calculated that *k* is 0.006, 0.023, 0.015 and 0.010 min^−1^ for unloaded AlPO_4_-5, Mo : Zn (2)/AlPO_4_-5, Mo : Zn (4)/AlPO_4_-5 and Mo : Zn (6)/AlPO_4_-5, respectively. It can be observed that *k* for Mo : Zn (2)/AlPO_4_-5 was the highest one; hence, this catalyst was the best one in our work.

### 3.6. Recyclability of the photocatalyst composite

**Fig. 11:**
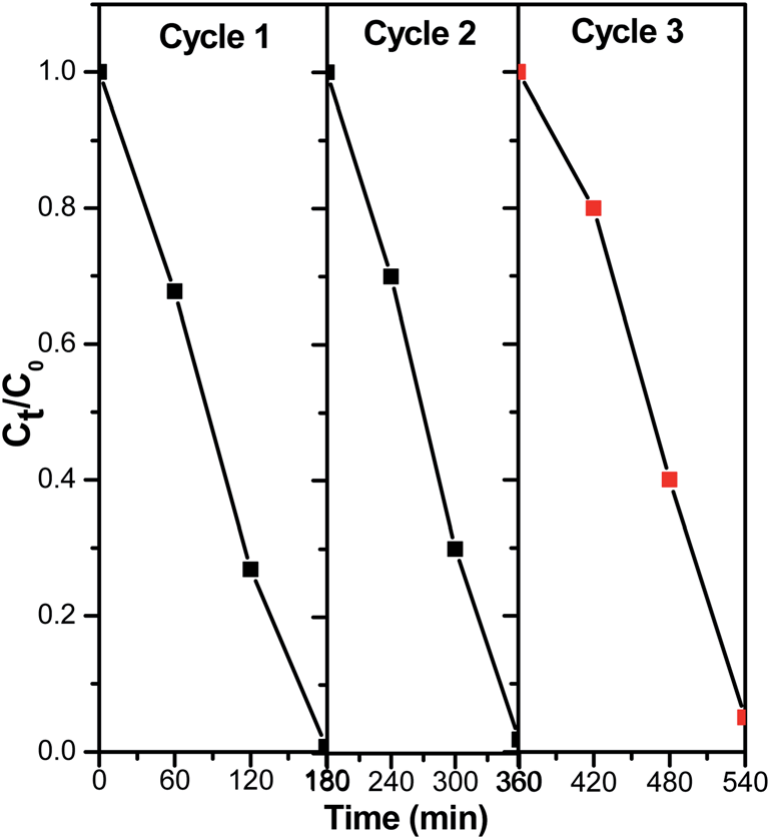
Recycling performance of ZnMoO_4_/AlPO_4_-5 with MZ (2) for PNP degradation in an aqueous solution. Reaction conditions: the dosage of catalysts 1.0 g l^−1^, initial PNP concentration of 0.04 g l^−1^ after 180 min irradiation time for each cycle.

The photocatalytic regeneration study aids in determining the economic value of the photocatalytic degradation process in terms of the produced catalyst's ability to be reused several times while simultaneously degrading organic contaminants (PNP). Herein, photodegradation processes, 1.0 g l^−1^ of catalyst and 0.04 g l^−1^ of PNP were stirred continuously in the cylindrical reactor with illuminate by pressure 125 W mercury lamp for 180 min. After that the solution was filtered and the filtrate was analyzed for PNP concentration. These catalyst was washed several times with double distilled water (DDW) to ensure complete removal of PNP or its intermediate and it was then treated with another amount of PNP solution. The results of the multiple photocatalytic cyclic test of ZnMoO_4_/AlPO_4_-5 with MZ (2) for PNP degradation in an aqueous solution is shown in Fig. 11. The results demonstrate the possibility of ZnMoO_4_/AlPO_4_-5 with MZ (2) for the regeneration process, with photocatalytic efficiency of 99.25%, 98.05% and 94.75% after 180 min for three successive photodegradation cycles, respectively. As clarified, there was insignificant decrease in catalytic performance which may cause due to photocatalyst mass loss during recycling studies. This issue can be resolved by encapsulating the photocatalysts in a solid substrate 20. These findings suggest that ZnMoO_4_/AlPO_4_-5 with MZ (2) has considerable promise as a photodegradation catalyst for the practical degradation of PNP in aqueous solutions.

### 3.7. Mechanism behind PNP by ZnMoO_4_/AlPO_4_-5

**Fig. 12:**
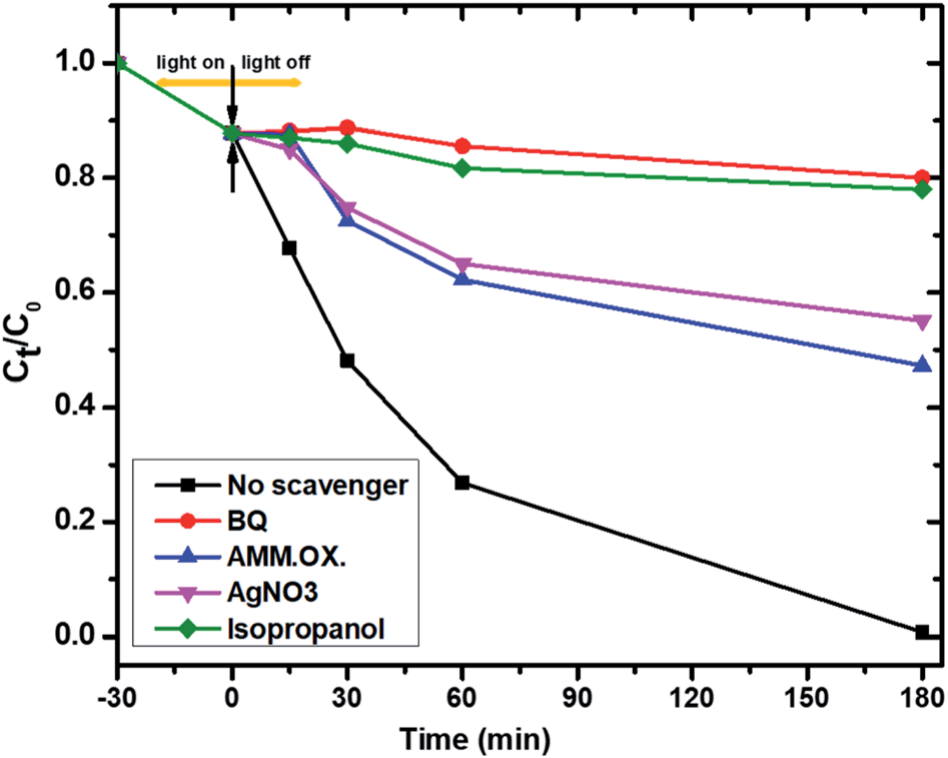
Inhibition of the photocatalytic degradation of PNP with different active-species scavengers BQ (for ˙O_2_^−^), IPA (for ˙OH), AO (for h^+^) and AgNO_3_ (for e^−^). Reaction conditions: the dosage of catalyst 1.0 g l^−1^, initial PNP concentration of 0.04 g l^−1^ after 180 min irradiation time.

To further study the photocatalytic mechanism and identify the main oxidative species (h^+^, e^−^, ˙O_2_^−^ and ˙OH) in the photocatalytic process, the effect of active-species scavengers on the photodegradation of PNP has been studied, and the results are presented in Fig. 12. Therefore, isopropyl alcohol (IPA), benzoquinone (BQ), ammonium oxalate (AO), and silver nitrate (AgNO_3_) were introduced as scavengers for hydroxyl radicals (˙OH), superoxide radicals (˙O_2_), holes (h^+^) and electrons (e^−^) respectively.^49^ As shown in Fig. 12, the photocatalytic degradation rates of PNP using Mo : Zn (2)/AlPO_4_-5 were decreased dramatically when BQ as an ˙O_2_^−^ and IPA as ˙OH scavengers were added into the photoreaction system.^50^ Conversely, the addition of AO and AgNO_3_ minimize the photocatalytic degradation of PNP using the same catalyst, suggesting that the h^+^ and e^−^ reactive species are only partially involved in the photocatalytic process. On the other hand, the degradation efficiencies decreased dramatically after adding BQ and IPA into the photoreaction system compared with no scavenger under similar conditions. Therefore, it can be concluded that:

(a) The ˙OH and ˙O_2_^−^ radicals are the predominant active species of the PNP degradation^51^ which chemically bonded interface structure between Mo, ZnO, and AlPO_4_-5 catalysts^52^ while,

(b) The dissolved O_2_ and H_2_O molecules significantly influence PNP photocatalytic degradation reaction. Thus, the influences of the scavengers on the reaction rate were in the order of the main active oxidation species:BQ (for ˙O_2_−) > IPA (for ˙OH) > AO (for h^+^) > AgNO_3_ (for e^−^).

**Scheme 1:**
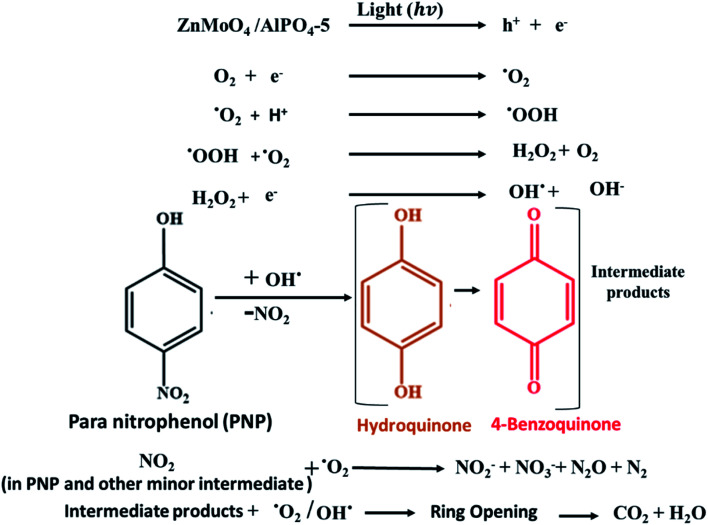
The proposed mechanism of the photocatalytic degradation of PNP on ZnMoO_4_/AlPO_4_-5 catalysts.

Consequently, the photodegradation of 4-nitrophenol with hydroxyl radicals and superoxide anion radicals produces hydroquinone and benzoquinone, which are then oxidized to produce intermediates containing a large number of ring-opening products. Finally, these intermediates will be oxidized to form CO_2_ and water. These intermediated was confirmed by HPLC results Fig. S3 and S4† validated with standards for the identification of compounds show that the main photo products of PNP degradation are acid products (RT = 2.5 min at *λ* = 280 nm), hydroquinone (RT = 3.6 min at *λ* = 280 nm) and Benzoquinone (RT = 4.3 min at *λ* = 280 nm), where RT is the retention time and *λ* is the wavelength. At the zero time, the degradation of PNP equals zero. After 5 min the rate of nitrophenol degradation, acid products, hydroquinone, and benzoquinone began to increase with time. After 180 min, the rate of degradation of phenol reached nearly to 100% where that of benzoquinone, hydroquinone and acid products began to disappear. These results revealed that hydroquinone and *p*-benzoquinone were the major intermediates in the reaction, which revealed that the hydroxylation occur at the *para* position mainly. Additionally, it was thought that the majority of the organic nitrogen in PNP was oxidised to NO_2_^−^ and NO_3_^−^, while the remaining organic nitrogen was transferred to N_2_, N_2_O, or smaller molecule organic nitrogen that was adsorbed or released into the air.^53^ As a result, Scheme 1 proposes the main degradation of PNP pathway.

## 4. Electrochemical measurements

The improvement of pristine AlPO_4_-5 as an electrode material for SC application depending on a co-impregnation method with different molar ratios of molybdenum and zinc bimetal supported AlPO_4_-5 (ZnMoO_4_/AlPO_4_-5) suggests a helpful strategy.

**Fig. 13:**
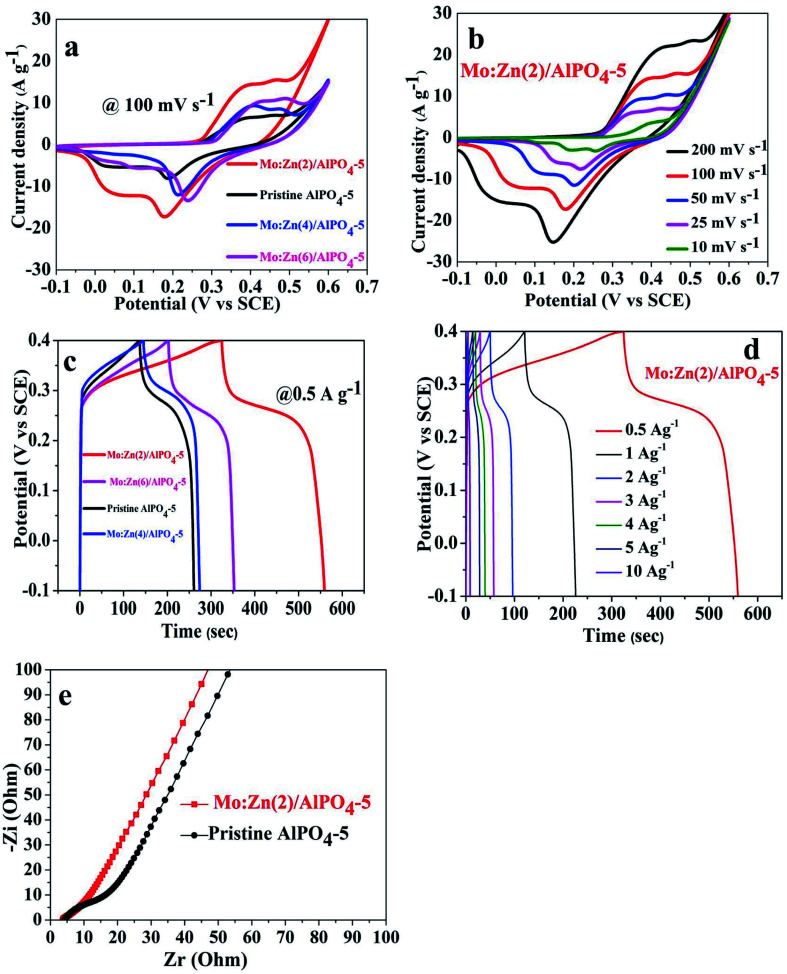
(a) CV curves of pristine AlPO_4_-5 and its corresponding Mo, Zn supported AlPO_4_-5 samples with different mole ratios, (b) CV curves Mo : Zn (2)/AlPO_4_-5 at different scan rates, (c) GCD curves of pristine AlPO_4_-5 and its corresponding Mo, Zn supported AlPO_4_-5 samples with different mole ratios at 0.5 A g^−1^, (d) CD curves Mo : Zn (2)/AlPO_4_-5 at different current densities, and (e) EIS spectrum of pristine AlPO_4_-5 and Mo : Zn (2)/AlPO_4_-5, respectively.

In this regard, the electrochemical responses of pristine AlPO_4_-5 and its corresponding molybdenum–zinc supported AlPO_4_-5 samples: Mo : Zn (2)/AlPO_4_-5, Mo : Zn (4)/AlPO_4_-5, and Mo : Zn (6)/AlPO_4_-5 were evaluated. Fig. 13a exhibited the CV curves of the pristine AlPO_4_-5 and its corresponding molybdenum–zinc supported AlPO_4_-5 samples with different ratios at a sweeping rate of 100 mV s^−1^. It is noticeable that the co-impregnation of Mo : Zn species on AlPO_4_-5 affected its integral CV area that witnessed from the increment in the integral current CV curve area, which generally increased by adding Mo and Zn due to an increase in the number of active redox sites. Here, Mo : Zn (2)/AlPO_4_-5 sample achieved the best integral CV area resulting in low charge transports resistance and low ion diffusion resistance, suggesting its higher capacitance. It is revealed that when Mo, Zn mole ratio content increased above 2, the electrochemical response was slightly declined but still more than the pristine AlPO_4_-5. This result may be ascribed to when Mo, Zn mole ration increased more than 2 will block some of the redox-active sites which restricted the interaction with the ionic electrolyte leading to the loss of its capacitance.^51,53–55^ As shown in (Fig. 13b), the CV curves of Mo : Zn (2)/AlPO_4_-5 were performed at different scan rates from 10 mV s^−1^ to 200 mV s^−1^ to evaluate its electrochemical performance as electroactive material for SC. The as-synthesized materials exhibited a prominent redox couple, suggesting its faradic-redox nature during the charge/discharge route, showing a typical battery-type material.^25^ By increasing the scan rate (Fig. 13b), the integral current CV area gradually increased, indicating low charge transfer and ion diffusion resistances. There is a slight deviation in the shape of CV curves at the low sweep rates, proposing the good reversibility of the redox reactions.^56^ At a high scan rate, the anodic and cathodic peaks are slightly shifted to more positive and more negative potentials, respectively, due to the polarization of electrode material.

Moreover, the GCD curves were performed within a potential window from 0 to 0.4 V as exhibited in (Fig. 13c and d). The as-synthesized Mo : Zn (2)/AlPO_4_-5 achieved the longest discharging time. Practically flat charge/discharge plateaus of the GCD curves are observed for all the samples at a constant voltage stage, showing a classic battery-type material consistent with the results obtained from the CV curves.

Based on eqn (1) and (2), the specific capacitances (*C*_sp_) (F g^−1^) and the specific capacities (*C*_s_) (C g^−1^) of Mo : Zn (2)/AlPO_4_-5 were calculated at diverse current densities depending on the corresponding GCD discharge curves. Hopefully, the as-synthesized Mo : Zn (2)/AlPO_4_-5 electrode exhibited *C*_sp_ of 725 F g^−1^ (290 C g^−1^), 328 F g^−1^ (164 C g^−1^), 150 F g^−1^ (60 C g^−1^), 87.5 F g^−1^ (35 C g^−1^), 62.5 F g^−1^ (25 C g^−1^), 50 F g^−1^ (20 C g^−1^), and 20 F g^−1^ (8 C g^−1^) at current densities of 0.5 A g^−1^, 1 A g^−1^, 2 A g^−1^, 3 A g^−1^, 4 A g^−1^, 5 A g^−1^, and 10 A g^−1^, respectively. Also, the calculated *C*_sp_ and its corresponding *C*_s_ for pristine AlPO_4_-5 and its corresponding molybdenum–zinc supported AlPO_4_-5 samples: Mo : Zn (2)/AlPO_4_-5, Mo : Zn (4)/AlPO_4_-5 and Mo : Zn (6)/AlPO_4_-5 at 0.5 A g^−1^ were 400 F g^−1^ (160 C g^−1^), 725 F g^−1^ (290 C g^−1^), 450 F g^−1^ (180 C g^−1^), and 481.25 F g^−1^ (192.5 C g^−1^), respectively.

EIS is considered a supportive method to explore the kinetic of electrons within redox reactions and ion diffusion. In this regard, (Fig. 13e) showed the Nyquist plot for pristine AlPO_4_-5 and Mo : Zn (2)/AlPO_4_-5 sample. The slope of the straight line represented the ion diffusion resistance in the low-frequency region. In the high-frequency region, the semicircle diameter indicated the charge transfer resistance that assesses the ease of electronic transportation within the redox reactions and the equivalent series resistance (ESR) obtained from the intersection of the curve at the high-frequency part. ESR suggests the summation of electrolyte resistances, active material, and current collector contact resistance.^57^ As exhibited in Fig. 13e, the ESR of pristine AlPO_4_-5 and Mo : Zn (2)/AlPO_4_-5 sample is not changed. Moreover, in the low-frequency part, the straight line of Mo : Zn (2)/AlPO_4_-5 electrode showed a comparatively more vertical line than that of the pristine sample, representing the co-impregnation of Mo and Zn reduced its ion diffusion resistance and charge transfer resistance.^58^ This result indicates that Mo : Zn (2)/AlPO_4_-5 is a promising material for SC applications.

## 5. Conclusion

In summary, we developed a simple strategy for synthesizing ZnMoO_4_/AlPO_4_-5 nanorod composites with molar ratios of Mo : Zn of 2, 4 and 6. The photocatalytic efficiency of the composites was determined by measuring the degradation of PNP under visible light irradiation. Notably, the ZnMoO_4_/AlPO_4_-5 composite with Mo : Zn molar ratios (2) exhibits the highest photocatalytic activity for PNP degradation. Additionally, supercapacitive tests on ZnMoO_4_/AlPO_4_-5 electrodes were conducted. This electrode has a maximum capacitance of 725 F g^−1^ at the current density of 0.5 A g^−1^ for Mo : Zn molar ratios (2). These results demonstrate that the prepared composites are excellent as light-driven photocatalytic materials and suitable for supercapacitor applications.

## Conflicts of interest

There are no conflicts to declare.

## Supplementary Material

RA-012-D2RA00268J-s001
