# Supplementary material for: Facile synthesis of ZnMoO4/AlPO4-5 nanorod composites as visible-light-driven photocatalysts and high-performance energy storage materials
Source: RSC Adv. 2022 Mar 2;12(12):7120–32. doi: 10.1039/d2ra00268j (PMC8982132; doi:10.1039/d2ra00268j)
Supplement: RA-012-D2RA00268J-s001 [file RA-012-D2RA00268J-s001.pdf]

Electronic Supplementary Material (ESI) for RSC Advances.
This journal is © The Royal Society of Chemistry 2022

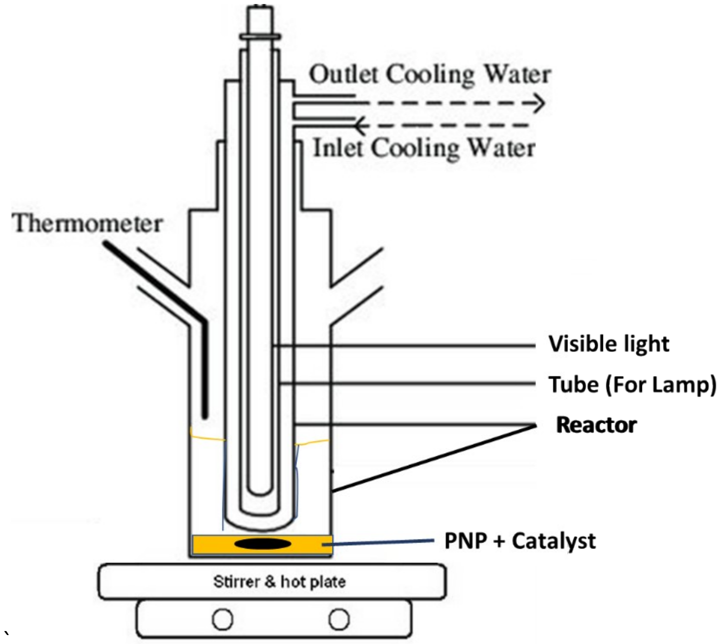

S1: Diagram of the photoreactor.

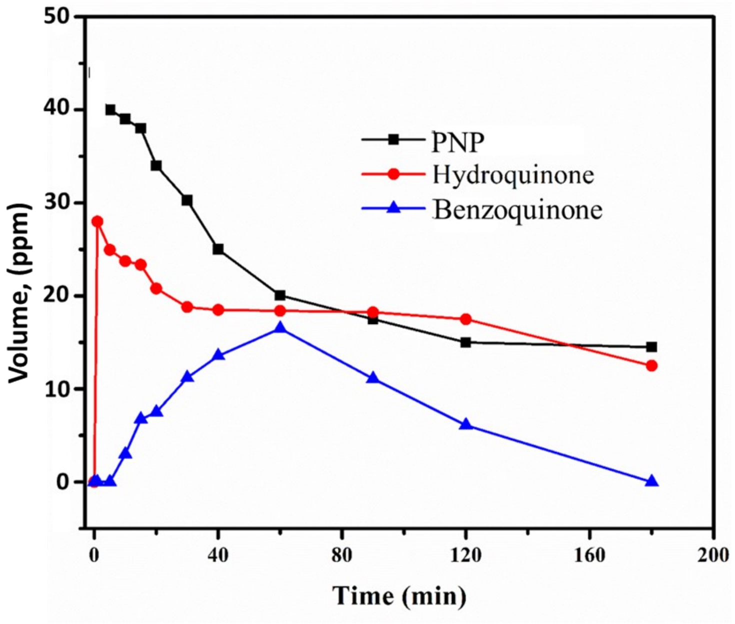

S2: Variation of Hydroquinone and 4-Benzoquinone (BZQ) as intermediate products concentration during the degradation of PNP

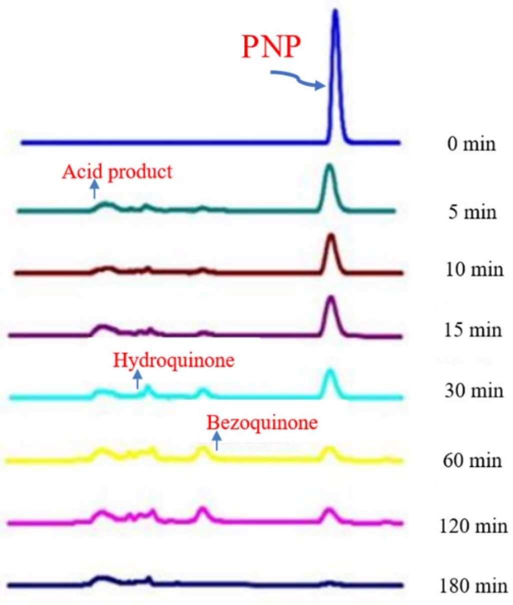

S3: HPLC chromatograms for PNP degradation at different times of irradiation.
